# Exercise rehabilitation in COPD and heart failure: comparison of two national audits

**DOI:** 10.1183/23120541.00131-2022

**Published:** 2022-11-28

**Authors:** Amy V. Jones, Rachael A. Evans, Alexander S. Harrison, Lauren B. Sherar, Michael C. Steiner, Patrick Doherty, Sally J. Singh

**Affiliations:** 1 Dept of Respiratory Sciences, University of Leicester, Leicester, UK; 2 Centre for Exercise and Rehabilitation Sciences, NIHR Leicester Biomedical Research Centre-Respiratory, Glenfield Hospital, University Hospitals of Leicester NHS Trust, Leicester, UK; 3 National Centre for Sports and Exercise Medicine, School of Sport and Exercise Sciences, Loughborough University, Loughborough, UK; 4 Dept of Health Sciences, University of York, York, UK

## Abstract

**Background:**

Pulmonary (PR) and cardiac rehabilitation (CR) are recommended in the management of chronic obstructive pulmonary disease (COPD) and chronic heart failure (CHF); the impact of coexisting COPD and CHF on completion and outcomes of rehabilitation programmes is unknown. We examined enrolment, completion and clinical outcomes of CR and PR in adults with COPD, CHF and coexisting COPD and CHF.

**Methods:**

The National Audit of CR and National COPD Audit Programme: clinical audits of PR were analysed (211 PR and 237 CR programmes); adults with a diagnosis of CHF, COPD or coexisting COPD and CHF were identified (COPD+CHF or CHF+COPD according to database). Propensity matching was conducted (age, sex, body mass index and functional status) between COPD+CHF and COPD, and CHF+COPD and CHF. Group by time interaction was examined using mixed 2×2 analysis of variance.

**Results:**

Those with CHF+COPD had lower enrolment and completion of CR compared to those with CHF; there were no differences in PR enrolment or completion between the two groups. Adults with COPD made a significantly larger gain in the incremental shuttle walk test compared to adults with COPD+CHF following PR (59.3 m *versus* 37.4 m); the improvements following CR were similar (CHF 77.3 m *versus* CHF+COPD 58.3 m). Similar improvements were made in the 6-min walk test following CR (CHF 45.1 m *versus* CHF+COPD 38.8 m) and PR (COPD 48.2 m *versus* COPD+CHF 44.0 m). Comparable improvements in quality of life and mood state were made following CR and PR, regardless of diagnosis.

**Conclusion:**

We have demonstrated that multi-morbid adults benefit from exercise-based rehabilitation, yet efforts are needed to promote completion. These findings support group-based, tailored, multi-morbid exercise rehabilitation.

## Abstract 2

**National audit data reveal adults with COPD, CHF and coexisting disease benefit from exercise-based rehabilitation. Effort is needed to improve completion of rehabilitation. The data support group-based, tailored, multimorbid exercise rehabilitation.** https://bit.ly/3yY6G9M

## Introduction

Chronic obstructive pulmonary disease (COPD) and chronic heart failure (CHF) are common long-term conditions; COPD is estimated to be the fourth leading cause of death worldwide [1] and there were an estimated 384 million cases of COPD in 2010 [2]. Similarly, CHF is extremely prevalent, with estimates suggesting 29 million people worldwide are affected [3], of which 920 000 people live within the UK [4]. Both COPD and CHF often result in individuals experiencing exertional dyspnoea and fatigue, with a reduced exercise capacity and quality of life [5, 6]. There are shared risk factors associated with CHF and COPD, including aging, cigarette smoke and inactivity [7, 8]. Due to these shared risk factors, adults with COPD often suffer from cardiac comorbidities [8] and cardiovascular disease is a leading cause of death in mild to moderate COPD [9]. The prevalence of coexisting COPD and CHF is high; estimates of comorbid COPD in adults with CHF ranges from 9 to 41% in European cohorts and 11–52% in North American cohorts [10]. A meta-analysis suggested that the prevalence of CHF in a COPD population ranged from 5 to 41% [11].

Exercise rehabilitation is recommended in the management of both COPD and CHF [12, 13]. Pulmonary rehabilitation (PR) is a multidisciplinary treatment that seeks to improve physical and psychological condition and promote long-term adherence to health-enhancing behaviours, whilst reducing disability and handicap in people with lung disease [14]. Benefits include improved quality of life as well as functional and maximal exercise capacity in adults with COPD [15]. Exercise-based cardiac rehabilitation (CR) favourably influences the underlying causes of cardiovascular disease and optimises a patient's physical, mental and social conditions [16]. Specifically, within the CHF population, CR reduces the risk of all-cause and CHF-specific hospital admissions in the short term (up to 12 months) and may lead to clinically important improvements in health-related quality of life compared to a no exercise control [17]. Due to the secondary-prevention nature of CR, the most common diagnosis and treatment groups are post myocardial infarction, percutaneous coronary intervention and coronary artery bypass graft [18].

Acknowledging the high prevalence of coexisting disease and the shared symptoms of breathlessness and fatigue, there is interest in combining exercise rehabilitation for adults with COPD and CHF [19–21]. The UK's National Health Service (NHS) supports the delivery of one exercise rehabilitation programme for adults presenting with breathlessness [22], and there is support for individualised multi-morbid exercise rehabilitation [23]. However, there is limited research examining clinical outcomes in adults with cardiorespiratory disease using national CR and PR audit data [24]. Furthermore, PR and CR programmes may view the presence of comorbidity differently, which may result in an enrolment and completion bias between those with cardiorespiratory disease or single cardiac/ pulmonary disease. Understanding this further may provide insight into how these patients travel through the UK healthcare system and the approach of PR and CR services, including the collection of appropriate outcome measures. Within the UK, two independent audit datasets for CR and PR have been established [25, 26], providing an opportunity to compare data from adults with cardiorespiratory disease and exercise-based rehabilitation, including enrolment and completion rates, and clinical outcome measures.

Therefore, using two national PR and CR audit datasets, the aim of this study is to compare the enrolment and completion rates, and examine the clinical outcomes of PR and CR in adults with COPD, COPD and comorbid CHF, CHF, and CHF and comorbid COPD, respectively.

## Material and methods

### Study subjects

PR services in England and Wales (n=230) were invited to participate in the National COPD Audit Programme: Clinical audit of Pulmonary Rehabilitation [25]. Caldicott Guardian approval was obtained by each PR programme and written informed consent was provided by each patient. The PR audit included all patients with a primary respiratory diagnosis of COPD that either attended their PR assessment or their first PR class between 12 January 2015 and 10 April 2015. Data were collected for a further 3 months (until 10 July 2015) to allow for PR completion and were entered *via* an online data collection tool. Approval from the Healthcare Quality Improvement Partnership was given for this analysis and a data sharing agreement was approved.

Data from CR services across England, Northern Ireland, Wales, the Isle of Man and the Channel Islands were inputted on the National Audit of Cardiac Rehabilitation (NACR) through NHS Digital [26]. NHS Digital allows NACR users to input patients’ data based on section 251 of the NHS Act 2006, regulated by the Health Research Authority's Confidentiality Group, which permits hospitals to collect identifiable patient data without the requirement for individual consent; patients were informed of the right to withdraw at any time. All NACR users are clinically approved and verified through a Caldicott Guardian. NHS Digital supplies the NACR with anonymised monthly downloads. Data were collected from all eligible patients who were offered CR from April 2007 until the end of November 2018, in line with the annual report [27]. This study was valid for NACR purposes and adheres to agreed data protection processes. Author A.V.J. received approval to utilise the NACR data.

Within the PR dataset, coexisting CHF was identified with the question “does the patient have any other significant medical conditions?” in which those that marked “left heart failure” were identified as COPD+CHF. Adults from the CR dataset were first identified if they had CHF (a primary diagnosis of CHF, an acute CHF event, previous diagnosis of CHF or a myocardial infarction with CHF) and then those with coexisting COPD (chronic bronchitis and emphysema) were either self-reported or confirmed using medical records (CHF+COPD).

### Study design

The main objective of this study was to examine enrolment, completion and outcomes of CR and PR in adults with COPD, CHF and coexisting disease.

### Methods

Assessment, enrolment and completion of PR and CR was examined. Within the PR dataset, the date the participant was assessed prior to beginning PR or the date of their first PR class was used as their date of assessment. The date of attending the first class of the programme was used as date of enrolment and completed refers to whether a discharge assessment was performed. Within the CR dataset, enrolment was defined as a participant having a full assessment of the CR core components [16] with agreed goals and targets and with a structured programme starting. Completion was confirmed where participants had a programme end date or a post-rehabilitation assessment with no reason for programme noncompletion stated.

A detailed description of the outcome measures analysed from the national CR and PR audit datasets is provided within the published audit annual reports [28, 29]. The CR and PR audit datasets both report the incremental shuttle walk test (ISWT) and the 6-min walk test (6MWT) [30, 31]. The minimum clinically important difference (MCID) for the ISWT and 6MWT following PR is between 35.0–36.1 m and 30 m accordingly [32, 33]. The MCID for the ISWT in adults with CHF following CR is 41.5 m, and 30.1 m in the 6MWT [34, 35].

Mood state and disease specific measures of quality of life were analysed using the Hospital Anxiety and Depression Scale [36] and the Minnesota Living with Heart Failure questionnaire [37] (CR dataset) and the Chronic Respiratory Questionnaire (CRQ) and [38] the COPD Assessment Test (CAT [39]) (PR dataset). Measures of cardiovascular risk (waist circumference, blood pressure, cholesterol, triglycerides and glycated haemoglobin) were analysed in the CR dataset.

### Analysis

Due to data restrictions, the CR and PR datasets were not combined, and analyses were undertaken for the two datasets in parallel. A valid case analysis was used in the CR dataset, ensuring only those with a confirmed presence or absence of chronic bronchitis and emphysema were included.

The distribution of baseline data was checked and appropriate parametric and nonparametric tests were used, with the mean (standard deviation) or median (interquartile range) reported. IBM SPSS versions 23, 24 and 25 were used and an alpha level ≤0.05 was considered to be statistically significant. A mixed 2×2 analysis of variance was used to assess differences between two groups (COPD *versus* COPD+CHF and CHF *versus* CHF+COPD) at two times (pre and post rehabilitation). Within-group effect size was calculated ((mean value post rehabilitation−mean value pre rehabilitation)/baseline sd)).

Adults with COPD+CHF and CHF+COPD were propensity matched 1:1 to those with COPD and CHF. Propensity matching was conducted using sex, age, body mass index (BMI) and a measure of functional status (Medical Research Council scale in the PR dataset and New York Heart Association scale in the CR dataset). Markers of disease severity (forced expiratory volume in the first second or ejection fraction) were not available, and therefore propensity matching using these variables was not possible. The statistical software R and the MatchIt package (versions 3.4.1 and 3.5.1) were used. Participants with missing data in one or more of the variables used to conduct propensity matching were removed from analyses.

## Results

A total of 211 programmes submitted data on 7413 participants with COPD to the PR dataset; 190 participants (2.6%) were identified as COPD+CHF. These participants were older, had a higher BMI, more likely to be male, had more comorbidities and had reduced exercise capacity compared to those with COPD alone (table 1). A total of 237 CR programmes uploaded data on 819 042 participants into the CR dataset, of which 6.4% (n=52 273) had CHF. Of those that had complete information regarding the presence or absence of comorbid COPD (64% n=33 437), 10.7% (n=3576) of adults had CHF+COPD. These adults were older, had a higher prevalence of osteoporosis, lower prevalence of diabetes and hypertension, and had reduced exercise capacity compared to those with CHF (table 1).

**TABLE 1:** Baseline demographics from completed pulmonary rehabilitation (PR) and cardiac rehabilitation (CR) audit datasets

	**PR**				**CR**			
	**n**	**COPD (n=7223)**	**n**	**COPD+CHF (n=190)**	**n**	**CHF (n=29 861)**	**n**	**CHF+COPD (n=3576)**
**Age (years)**	7222	70.0 (64.0–76.0)	190	74.0 (68.0–80.0)*	29 861	72.0 (62.0–80.0)	3576	73.0 (66.0–79.0)*
**BMI (kg·m^−2^)**	4775	26.8 (22.9–31.1)	123	27.8 (24.3–34.0)*	13 769	28.1 (24.9–32.4)	1501	27.9 (24.3–32.6)*
**Sex (male, %)**	7223	3817 (52.8)	190	131 (68.9)*	29 638	20 168 (68.0)	3558	2442 (68.6)
**Ethnicity (White British, %)**	7223	6357 (88.0)	190	166 (87.4)	29 861	21 181 (86.2)	3576	2552 (91.2)*
**Smoking status**	7056		186		12 896		1507	
Current (%)		1585 (22.5)		29 (15.6)		1148 (8.9)		276 (18.3)*
Former (%)		5038 (71.4)		141 (75.8)		6336 (49.1)		1032 (68.5)
Never (%)		433 (6.1)		16 (8.6)		5412 (42.0)		199 (13.2)
**MRC scale**	7223	3 (3–4)	190	4 (3–4)*	NA			
**NYHA scale**	NA				2891	2 (2–3)	359	2 (2–3)*
**Diabetes (%)**	7223	940 (13.0)	190	47 (24.7)*	29 861	9286 (31.1)	3576	1041 (29.1)*
**Stroke (%)**	7223	286 (4.0)	190	13 (6.8)	29 861	2753 (9.2)	3576	329 (9.2)
**Osteoporosis (%)**	7223	550 (7.6)	190	17 (8.9)	29 861	803 (2.7)	3576	133 (3.7)*
**Hypertension (%)**	7223	2162 (29.9)	190	72 (37.9)*	29 861	14 916 (50.0)	3576	1599 (44.7)*
**ISWT (m)**	3723	199.9 (134.4)	96	154.2 (103.5)*	2162	277.2 (152.6)	211	225.9 (138.8)*
**6MWT (m)**	2789	249.5 (114.9)	74	194.8 (101.0)*	2556	249.5 (119.3)	343	217.7 (105.0)*

*: p≤0.05 Data presented as mean (standard deviation), frequency (%) or median (interquartile range). 6MWT: 6 min walk test; BMI: body mass index; CHF: chronic heart failure; COPD: chronic obstructive pulmonary disease; ISWT: incremental shuttle walk test; MRC: Medical Research Council dyspnoea scale; NA: not applicable; NYHA: New York Heart Association.

**FIGURE 1:**
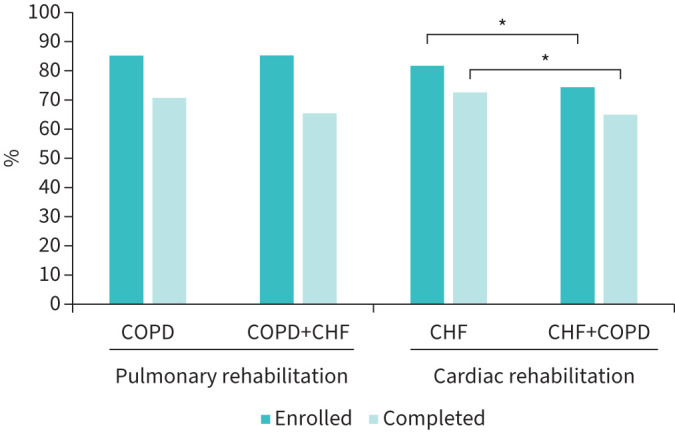
Percentage of adults that enrolled and completed pulmonary rehabilitation (PR) and cardiac rehabilitation (CR). *: p≤0.05. Percentages calculated from those that were assessed for PR or CR. CHF: chronic heart failure; COPD: chronic obstructive pulmonary disease.

Following PR assessment in those with COPD, 85.2% (n=6157) enrolled and of those 70.7% (n=4350) completed rehabilitation (figure 1). All adults with COPD+CHF were assessed (n=190), 85.3% (n=162) enrolled and, of those enrolled, 65.4% (n=106) completed the programme (figure 1). There was no difference in the PR enrolment (p=1.00) or completion rates (p=0.23) between the two groups.

Data from the CR audit revealed 62.6% (n=18 700) of adults with CHF that were referred to CR were assessed. Of those, 81.7% (n=15 271) enrolled into the programme and 72.6% completed it (n=11 081) (figure 1). Over half (58.5%, n=2091) of the adults with CHF and coexisting COPD that were referred to CR were assessed and, of those, 74.3% (n=1553) enrolled into the programme and 65% (n=1009) completed it (figure 1). A significantly lower number of adults with CHF+COPD enrolled (p<0.05) and completed CR (p<0.05) compared to adults with CHF only (figure 1).

Propensity matching resulted in 232 adults matched within the PR dataset and 612 adults matched within the CR dataset. Propensity-matched baseline data in all those that completed PR and CR is presented in supplementary table S3. After matching, there were no significant differences between the groups in age, BMI, sex, ethnicity and the presence of other coexisting health conditions. Statistical differences in smoking status remained between adults with CHF and CHF+COPD. Propensity matching did not alter statistical findings regarding enrolment or completion of PR or CR in these populations.

A statistically significant main effect for time revealed that on average, exercise capacity (ISWT) improved following PR and CR. A larger gain was made by those with COPD (59.3 m) compared to those with COPD+CHF (37.4 m) and the improvements made by adults with CHF and CHF+COPD were comparable (CHF 77.3 m; CHF+COPD 58.3 m) (table 2). The improvements made in the 6MWT following PR and CR were comparable in all participants (p>0.05) (table 2).

**TABLE 2:** Propensity-matched pre and post data from pulmonary rehabilitation (PR) and cardiac rehabilitation (CR)

	**PR**							
	**COPD (n=116)**			**COPD+CHF (n=116)**				
	**n**	**Pre**	**Post**	**Effect size**	**n**	**Pre**	**Post**	**Effect size**
**ISWT (m)**	41	173.9 (135.8)	233.2 (151.6)*	0.4	39	163.0 (115.4)	200.4 (128.4)*	0.3
**6MWT (m)**	19	233.0 (97.8)	281.2 (101.9)*	0.5	22	220.0 (89.8)	264.0 (110.0)*	0.5
**CRQ**–**dyspnoea**	33	2.7 (1.5)	3.6 (1.4)*	0.6	30	2.7 (1.1)	3.6 (1.4)*	0.8
**CRQ–fatigue**	33	3.8 (1.4)	4.4 (1.4)*	0.4	30	3.2 (1.5)	3.9 (1.4)*	0.5
**CRQ–emotion**	33	4.4 (1.3)	5.1 (1.2)*	0.5	30	4.5 (1.4)	5.1 (1.4)*	0.4
**CRQ–mastery**	33	4.5 (1.3)	5.2 (1.4)*	0.5	30	4.2 (1.4)	5.2 (1.5)*	0.7
**CAT**	38	21.0 (8.3)	15.9 (7.4)*	0.6	31	21.1 (8.1)	17.3 (7.5)*	0.5

	**CR**							
	**CHF (n=306)**			**CHF+COPD (n=306)**				
	**n**	**Pre**	**Post**	**Effect size**	**n**	**Pre**	**Post**	**Effect size**
**ISWT (m)**	26	239.2 (90.6)	316.5 (109.6)*	0.9	17	293.5 (154.6)	351.8 (187.3)*	0.4
**6MWT (m)**	64	248.7 (124.7)	293.8 (102.1)*	0.4	45	247.8 (119.4)	286.6 (98.6)*	0.3
**WC (cm)**	64	101.6 (15.7)	101.9 (15.9)	0.02	34	106.4 (14.3)	104.7 (14.6)*	0.1
**Systolic BP (mmHg)**	172	126 (22.2)	126 (21.0)	0.02	122	121 (19.4)	122 (18.1)	0.1
**Diastolic BP (mmHg)**	173	70 (12.6)	69 (10.5)	0.1	122	68 (9.9)	69 (11.2)	0.1
**Total cholesterol (mmol·L^−1^)**	48	4.4 (1.12)	4.0 (0.84)	0.4	31	4.2 (1.51)	4.1 (1.25)	0.1
**LDL (mmol·L^−1^)**	22	2.5 (0.85)	2.1 (0.67)	0.4	13	2.1 (0.77)	2.4 (0.97)	0.3
**HDL (mmol·L^−1^)**	33	1.2 (0.36)	1.3 (0.39)	0.1	19	1.2 (0.40)	1.2 (0.36)	0.1
**HDL:LDL**	27	3.8 (1.04)	3.6 (1.04)	0.2	15	3.5 (0.93)	3.7 (1.19)	0.2
**TG (mmol·L^−1^)**	23	1.3 (0.46)	1.3 (0.45)	0.00	14	1.6 (0.92)	1.6 (0.72)	0.00
**HbA1c (%)**	11	48.6 (10.78)	46.6 (8.31)	0.2	8	55.6 (19.87)	47.4 (10.91)	0.4
**HADS–anxiety**	138	5.7 (4.4)	4.9 (3.9)*	0.2	82	5.6 (3.6)	5.3 (3.5)	0.1
**HADS–depression**	138	6.1 (3.8)	4.6 (3.5)*	0.4	82	6.0 (3.6)	4.9 (3.3)*	0.3
**Minnesota**	54	39.8 (20.4)	33.5 (21.5)*	0.3	32	39.0 (24.3)	32.3 (19.6)*	0.3

*: p≤0.05. Data presented as mean (standard deviation). 6MWT: 6-min walk test; BP: blood pressure; CAT: COPD Assessment Test; CHF: chronic heart failure; COPD: chronic obstructive pulmonary disease; CRQ: Chronic Respiratory Questionnaire; HADS: Hospital Anxiety and Depression Scale; HbA1c: glycated haemoglobin; HDL: high-density lipoprotein; ISWT: incremental shuttle walk test; LDL: low-density lipoprotein; Minnesota: Minnesota Living with Heart Failure questionnaire; NA: not applicable; TG: triglycerides; WC: waist circumference.

A similar percentage of participants achieved the MCID in the 6MWT (COPD 79% (n=15); COPD+CHF 68% (n=15)), yet significantly more adults with COPD (71% (n=29) achieved the MCID on the ISWT compared to adults with COPD+CHF (46% (n=18)) following PR. The percentage of participants that achieved the MCID in the ISWT (CHF 69% (n=18); CHF+COPD: 59% (n=10)) and 6MWT (CHF 53% (n=34); CHF+COPD 45% (n=20)) was similar following CR.

There was a significant main effect of time for each domain of the CRQ, suggesting quality of life improved following PR in all participants (table 2). There were no differences in the magnitude of improvement made over time in the CRQ between adults with COPD and COPD+CHF. Quality of life appeared to significantly improve in all participants following CR, and there was no significant group by time interaction (table 2). Mean anxiety and depression scores decreased following CR and there were no group by time interactions (table 2). Symptom burden significantly reduced followed PR, yet there were no differences in the improvement made over time between adults with COPD and COPD+CHF (table 2). Upon completion of CR, there appears to be no change in any of the cardiometabolic variables (table 2).

## Discussion

This analysis suggests the process and clinical outcomes of CR and PR in adults with cardiorespiratory disease or single cardiac/pulmonary disease are similar, suggesting benefits are achieved from both CR and PR in this unique group of participants.

Results demonstrated an improved quality of life following PR and CR in this combined cardiorespiratory population, showing outcomes are not compromised by comorbid CHF or COPD; data is equivalent to single-disease audit data. An improved quality of life following PR is supported by a meta-analysis, in which all four domains of the CRQ improved more than the MCID [15, 40]. Symptom burden also improved following PR and the degree of improvement was similar in those with a single disease and those with comorbid cardiorespiratory disease. It is well established that the CAT result improves following PR [41]; however, to the best of our knowledge, this is the first study to analyse the CAT within PR in a comorbid COPD and CHF sample. Anxiety and depression significantly decreased following CR and there was no difference in the magnitude of change between adults with CHF and CHF and coexisting COPD; there is little published data examining the impact of CR on anxiety and depression in a CHF population.

The current analysis suggests a disparity may exist between enrolment and completion rates for CR and PR in adults with cardiorespiratory disease. Measures have been implemented nationally to increase enrolment of adults with CHF into CR, such as key performance indicators and a minimum standard stating that CR is offered to priority groups, including people with CHF [42]. However, it is widely acknowledged that the uptake of adults with CHF into CR is still too low [43]. There are a range of reasons as to why this occurs; adults with CHF present differently to the conventional post-myocardial infarction population, with a reduced exercise capacity and different educational needs [44]. They also present with a significant respiratory comorbidity, breathlessness, that often manifests itself during the exercise component of CR. It could be suggested that some staff may feel less experienced supporting this group of adults. The findings from the current analysis strengthen the rationale for developing a combined exercise-based rehabilitation service, delivered by a workforce trained to rehabilitate multi-morbid patients, that have sufficient capacity to enrol target populations.

A key finding from this analysis is the distinct lack of outcome measure data collected within CR; only 16% of adults with CHF or CHF+COPD had a measure of baseline exercise capacity, compared to 90% of those with COPD or COPD+CHF from PR. An assessment of exercise capacity is fundamental in prescribing individual exercise and maximising the potential gains that can be achieved [45]; an assessment of exercise capacity should be encouraged. This is of particular importance to the CHF population as regards the role of exercise training and symptom management. Analysis suggests markers of cardio-metabolic health were not impacted following CR in the CHF populations, which is of note given that reducing cardiovascular risk is a main objective of CR. This further supports the need for accurate exercise testing to ensure individualised exercise prescription is delivered. The lack of outcome data could be due to workforce training or competency, capacity issues, or difficulties uploading data into audit datasets. Regardless, efforts are needed to ensure these outcome measures are collected at baseline and upon programme completion.

The prevalence of cardiorespiratory diagnosis in the current analysis is potentially under-reported when compared to known prevalence estimates [10, 11]. This could be in part due to a lack of identification or management of the comorbid condition, as the referring healthcare professional may be respiratory or cardiac focused, and the disease diagnostics may not be completed. These low rates could also be due to an increased number of disease exacerbations in this group, which may impact referral. Efforts are needed to ensure this group of multi-morbid adults are identified and referred into exercise rehabilitation.

At present, there are no specific guidelines advising to which exercise-based rehabilitation programme an adult with multi-morbid cardiorespiratory disease should be referred. However, we have demonstrated that, regardless of comorbid cardiorespiratory disease, this population should be encouraged to complete exercise-based rehabilitation as benefits are still gained. Many CR and PR attendees are multi-morbid and present with generic symptoms, such as breathlessness. Furthermore, the CHF population attending CR is small and the programme is largely focussed on secondary prevention as opposed to CHF management; these patients may benefit from attending a generic exercise-based rehabilitation programme that consists of appropriate exercise testing and prescription. The standardising of outcome measures including nonspecific symptoms and generic quality of life would allow comparisons both within and between exercise-based rehabilitation programmes, and between various “disease-specific” exercise-based rehabilitation programmes. There is growing support for the amalgamation of cardiorespiratory rehabilitation services within the UK [19–22] and the creation of personalised multi-morbidity rehabilitation [23].

The interrogation of national audit data allows conclusions to be made from clinical rehabilitation programmes across the UK, as it reflects real-life data; we can be confident that the findings from the present analysis are generalisable and representative of UK clinical services. Furthermore, the analysis of these two datasets provided a unique opportunity to explore exercise-based rehabilitation in adults with cardiorespiratory disease. It is possible that a small number of participants were included in both datasets if they enrolled into CR and PR. The validity and accuracy of the comorbidity identification is also unknown; it was decided that adults with coexisting disease that were referred to CR had CHF as the index condition, and those referred to PR had COPD as the index condition. This assumption may not be robust as the degree of organ severity was not assessed, and it could be suggested that the population of adults with coexisting COPD and CHF are similar, regardless of their primary index disease and the rehabilitation programme attended. Large variations in sample size were present in the current analysis and this may explain the findings of statistical significance. Lastly, due to data governance restrictions, it was not possible to merge the CR and PR dataset, preventing statistical comparisons.

Overall, this study demonstrates the similar process and clinical outcomes of CR and PR in adults with cardiorespiratory disease, either as single or multi-morbid disease. However, efforts are needed to ensure multi-morbid cardiorespiratory disease is diagnosed within the rehabilitation populations and that appropriate exercise testing occurs, particularly within CR programmes. The clinical implications for cardiorespiratory exercise rehabilitation services following this analysis include prioritising the referral of adults with comorbid COPD and CHF into exercise rehabilitation and supporting a symptom-based model of rehabilitation, in this case breathlessness.

## Supplementary material

10.1183/23120541.00131-2022.Supp1 **Please note:** supplementary material is not edited by the Editorial Office, and is uploaded as it has been supplied by the author.
Supplementary material 00131-2022.SUPPLEMENT
