# Supplementary material for: Exercise rehabilitation in COPD and heart failure: comparison of two national audits
Source: ERJ Open Res. 2022 Nov 28;8(4):00131-2022. doi: 10.1183/23120541.00131-2022 (PMC9703148; doi:10.1183/23120541.00131-2022)
Supplement: Supplementary file 1 [file 00131-2022.SUPPLEMENT.pdf]

SUPPLEMENTARY MATERIAL

Supplementary Table 1: Propensity matched baseline demographics of PR and CR datasets

	Pulmonary Rehabilitation		*Cardiac Rehabilitation*	
	**COPD (n=116)**	**COPD + CHF (n=116)**	**CHF (n=306)**	**CHF + COPD (n=306)**
Age (years)	74.0 (67.0-80.0)	73.0 (68.0-79.8)	73.0 (65.0-80.0)	74.0 (66.0-78.0)
BMI (kg/m^2)	28.3 (24.3-32.6)	27.1 (24.1-34.0)	28.0 (24.6-32.4)	27.8 (24.4- 33.0)
Sex (male %)	84 (72.4)	86 (74.1)	233 (76.1)	228 (74.5)
Ethnicity (White British %)	99 (85.3)	102 (87.9)	239 (78.1)	248 (81.0)
MRC	4 (3-4)	4 (3-4)	N/A	
NYHA scale	N/A		2 (2-3)	2 (2-3)
Smoking status				
Current (%)	25 (22.1)	15 (12.9)	17 (5.7)	50 (16.9)
Former (%)	84 (74.3)	94 (81.0)	157 (52.7)	201 (68.1)
Never (%)	4 (3.5)	7 (6.0)	124 (41.6)	44 (14.9)
Diabetes (%)	20 (17.2)	28 (24.1)	94 (30.7)	96 (31.4)
Stroke (%)	5 (4.3)	9 (7.8)	28 (9.2)	27 (49.1)
Osteoporosis (%)	7 (6.0)	13 (11.2)	12 (3.9)	13 (4.2)
Hypertension (%)	44 (37.9)	43 (37.1)	138 (54.9)	128 (48.1)

Data presented as mean(SD), frequency (%) or median(IQR) BMI= body mass index; MRC= Medical Research Council dyspnoea scale, NYHA= New York Heart Association
